# 118. Feasibility of a Proactive Amoxicillin Oral Challenge Program for Inpatients with Penicillin Allergy at the Miami VAMC

**DOI:** 10.1093/ofid/ofab466.320

**Published:** 2021-12-04

**Authors:** Michael J Piazza, Paola Lichtenberger, Lauren Bjork, Alex Lazo-Vasquez, Minh Hoang, Viviana Temino

**Affiliations:** 1 University of Miami Hospital / Jackson Memorial, Ocean, New Jersey; 2 University of Miami Miller School of Medicine and the Miami VA Healthcare System and University of Miami, Miami, FL; 3 Miami Veterans Affairs Healthcare System, Miami, Florida; 4 Miami VA Medical Center, Miami, Florida

## Abstract

**Background:**

Ninety percent of patients who report penicillin (PCN) allergy are not truly allergic. Penicillin skin testing (PST) followed by oral challenge (OC) with amoxicillin (AMX) can evaluate unconfirmed PCN allergy. PST is taxing and requires trained staff, while OC is an acceptable alternative in patients with low-risk histories, who can safely undergo OC without PST. OC is performed in the outpatient Miami Veterans Affairs Medical Center (MVAMC) setting. Collaboration between Allergy, Antimicrobial Stewardship Program (ASP), and Hospital Medicine identified patients with low-risk histories and offered OC to inpatients.

**Methods:**

A daily report of MVAMC inpatients with PCN allergy was reviewed for appropriateness of OC (Fig 1). Hospice patients and those medically unstable or unable to consent were excluded. Appropriate consenting patients were challenged with AMX 500mg PO and observed for 60 minutes. If no reaction resulted, the PCN allergy label was removed. Epinephrine and diphenhydramine were available in case of adverse reaction. Those who were not OC candidates were offered outpatient PST (Fig 1).

Figure 1. Penicillin allergy history evaluation algorithm

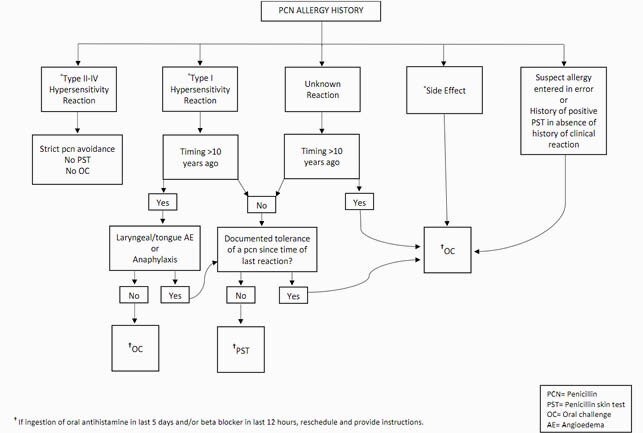

**Results:**

We evaluated 39 inpatients with PCN allergy from 3/10 - 5/27/21. Median age was 68 years; 94.9% were male (Table 1). The most common recorded reaction was unknown (Table 2). Thirteen (33.3%) did not qualify for OC, 7 (17.9%) refused, 2 (5.1%) were receiving a penicillin-derivative, 1 (2.6%) patient’s primary team refused consult, 2 (5.1%) patients were discharged prior to OC. Fourteen (38%) patients underwent OC with 0 adverse reactions; 0 patients required epinephrine or diphenhydramine. After OC, 5 patients had changes to their antibiotic regimen as a result of a negative OC. Limitations included 5 patients on beta-blockers, and 5 patients unable to consent.

Table 1. Demographics of Evaluated Inpatients, N = 39 (%)

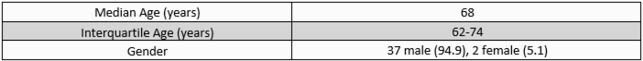

Note that 1 patient out of the 39, underwent DPC with cefpodoxime 200mg PO instead of amoxicillin for a reported allergy to ceftriaxone.

Table 2. Reported Reactions, N = 41 (%)

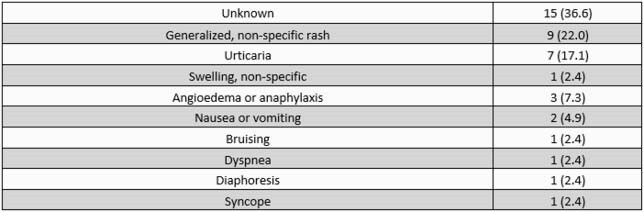

Total N exceeds evaluated patient number as one patient reported multiple reactions to receiving penicillin.

**Conclusion:**

Removing unnecessary PCN allergy labels using inpatient OC with AMX is safe and effective for those with low-risk allergy histories. Zero patients undergoing OC developed a reaction, suggesting that OC may be safely performed per our algorithm. Our protocol does not require specialized training and is reproducible in settings without an Allergy specialist. In the 3 months prior to this program there were 0 inpatient consults to evaluate PCN. Future plans include forming a multidisciplinary consult service.

**Disclosures:**

**All Authors**: No reported disclosures

